# DcR3 gene polymorphisms are associated with sporadic breast infiltrating ductal carcinoma in Northeast Chinese women

**DOI:** 10.18632/oncotarget.11153

**Published:** 2016-08-09

**Authors:** Zhenkun Fu, Shuang Chen, Shengwei Liu, Shaoli Han, Xiang Gao, Dalin Li, Dianjun Li

**Affiliations:** ^1^ Heilongjiang Provincial Key Laboratory for Infection and Immunity, Harbin Medical University and Heilongjiang Academy of Medical Science, 150081 Harbin, China; ^2^ Department of Immunology, Harbin Medical University and Heilongjiang Academy of Medical Science, 150081 Harbin, China; ^3^ Department of Breast Surgery, The Third Affiliated Hospital of Harbin Medical University, 150081 Harbin, China

**Keywords:** DcR3, TNFRSF6β, breast infiltrating ductal carcinoma, single nucleotide polymorphism, haplotype

## Abstract

Decoy Receptor 3 (DcR3), also called TNFRSF6β, is a member of the tumor necrosis factor receptor superfamily and is a soluble receptor for FasL. DcR3 is overexpressed in cancers and contributes to tumorigenesis through immune suppression and promotion of angiogenesis. We found that DcR3 is overexpressed in breast infiltrating ductal carcinoma (IDC) cells as compared with normal controls. We also conducted a case-control study analyzing associations of DcR3 polymorphisms with breast IDC risk. Subjects included 531 females with breast IDC and 592 age-matched healthy controls. Four DcR3 single nucleotide polymorphism loci with minor frequencies of more than 5% (rs3208008, rs41309931, rs2297441 and rs1291207) were genotyped using polymerase chain reaction restriction fragment length polymorphism and sequencing. Our results revealed significant differences in rs41309931genotypes and alleles (*P* < 0.01). Based on Haploview software analysis, the haplotype block A_rs3208008_ G_rs41309931_ G_rs2297441_ A_rs1291207_ exhibited the highest frequency, but, haplotype blocks A_rs3208008_ T_rs41309931_ G_rs2297441_ A_rs1291207_ and C_rs3208008_ G_rs41309931_ G_rs2297441_ A_rs1291207_ were associated with breast IDC risk. This study also detected associations between DcR3 gene polymorphisms and the clinicopathological features of breast IDC, including lymph node metastasis and C-erbB2, P53, estrogen receptor and progesterone receptor status. These data indicate that DcR3 gene polymorphisms are associated with sporadic breast IDC risk in Northeast Chinese females.

## INTRODUCTION

Decoy Receptor 3 (DcR3), which maps to chromosomal region 20q13.3, is also called tumor necrosis factor receptor super family member 6β (TNFRSF6β) and can bind FasL, LIGHT and TL1A [1, 2]. As a soluble protein, DcR3 can be secreted because it lacks transmembrane and intracellular localization peptides. LIGHT, TL1A and their signal receptors are essential for promoting T cell proliferation, and DcR3-mediated blockade of these ligands was shown to reduce T cell activity [3, 4]. DcR3 can modulate activation and differentiation of dendritic cells [5] and macrophages [6, 7]. DcR3 expression is few detectable in normal tissues, but is upregulated in some malignant tissues [8–11]. Thus, DcR3 may be a potential prognostic indicator or marker for early tumor detection in certain kinds of cancer.

Infiltrating ductal carcinoma (IDC) is the most common type of breast cancer and accounts for about 80% of cases [12]. Both natural and adaptive immune responses can influence breast cancer occurrence, development and prognosis [13]. Furthermore, co-stimulatory or co-inhibitory molecules are important second signal factors in activation or inhibition of the adaptive immune response. As a co-inhibitory molecule, DcR3 may play a crucial role in breast IDC. In recent years, aberrant DcR3 expression was observed in breast cancer cells and in the breast cancer cell line, MCF-7 [14, 15].

Single nucleotide polymorphisms (SNPs), as natural sequence variations, may lead to differential DcR3 expression. With respect to genetic variation, SNPs have been utilized in breast cancer research [16, 17]. To data, the DcR3 pathway has primarily been associated with autoimmune diseases [18]. We performed a case-control study to determine whether DcR3 gene region SNPs impact susceptibility to breast IDC. We established an association between DcR3 gene polymorphisms and breast IDC risk in Chinese Han females in Heilongjiang Province (CHB population). We also assessed the relationship between DcR3 polymorphisms and breast IDC prognostic factors, such as estrogen receptor (ER), progesterone receptor (PR), P53 and C-erbB-2 levels.

## RESULTS

### DcR3 expression in breast IDC tissue and breast cancer cell lines

We found that DcR3 was overexpressed in tumors as compared to pericarcinomatous tissues (*P* = 0.0001). DcR3 expression was positive in 76.4% of tumors and 40.0% of pericarcinomatous tissues (Supplementary File A). Thirty breast IDC tissue samples exhibited positive ER, PR and HER2 (human epidermal growth factor receptor-2) expression. There was no significant relationship between DcR3 and ER positivity in the tumor samples, and we also found the similar results for PR and HER2 (Supplementary Figure S1A).

Western blotting showed that all three breast cancer cell lines expressed higher DcR3 levels than HMECs, with no expression difference between the cancer cell lines (Supplementary Figure S1B).

### Genotypes and alleles

A total of 531 breast cancer patients and 592 healthy controls were successfully genotyped in this study. All samples were from females of the same geographical region with Chinese Han nationality. Cases and controls were age-matched and age did not affect breast IDC risk by logistic regression analysis. Because we ran out of DNA samples, the missing rs2257440 and rs909341 frequencies were more than 25%, and so these two SNP loci were not included in the statistical analysis. All of the other four DcR3 SNPs were in accordance with Hardy-Weinberger equilibrium in cases and controls.

Genotype and allele frequencies are shown in Tables 1 and 2. We found no significant differences in rs3208008 in the 5′-near gene region, rs2297441 in the 5′-UTR and rs1291207 in an intron (*P* > 0.05). rs41309931 in the 5′-near gene region (additive *P* = 0.002, dominant *P* = 0.0005) was associated with breast IDC. In the allele analysis, the T allele in rs41309931 (*P* = 0.001) had lower frequencies in tumors. We corrected the *P-value* in this allele for multiple testing by using 10,000 permutations in the Haploview program (*P* = 0.0033).

**Table 1:** Genotype frequencies of DcR3 polymorphisms and their associations with breast cancer risk

SNP	Minor, (a)	Major, (A)	Cases (%)			Controls (%)			*P* value for model of inheritance		
			‘AA’	‘Aa’	‘aa’	‘AA’	‘Aa’	‘aa’	Additive	Dominant	Recessive
Rs3208008	C	A	249 (47.52%)	226 (43.13%)	49 (9.35%)	285 (48.80%)	248 (42.47%)	51 (8.73%)	0.887	0.670	0.720
Rs41309931	T	G	298 (57.09%)	189 (36.21%)	35 (6.70%)	269 (46.54%)	259 (44.81%)	50 (8.65%)	**0.002**	**0.0005label^#^**	0.228
Rs2297441	A	G	259 (49.81%)	226 (43.46%)	35 (6.73%)	267 (46.11%)	266 (45.94%)	46 (7.95%)	0.426	0.221	0.442
Rs1291207	G	A	263 (50.29%)	203 (38.81%)	57 (10.90%)	270 (46.39%)	249 (42.78%)	63 (10.83%)	0.381	0.196	0.969

Rs3208008 cases *n* = 524, missing *n* = 7; controls *n* = 584, missing *n* = 8.

Rs41309931 cases *n* = 522, missing *n* = 9; controls *n* = 578, missing *n* = 14.

Rs2297441 cases *n* = 520, missing *n* = 11; controls *n* = 579, missing *n* = 13.

Rs1291207 cases *n* = 523, missing *n* = 8; controls *n* = 582, missing *n* = 10.

Minor allele ‘a’ and the major ‘A’ are shown in the table. ‘AA’, ‘Aa’, ‘aa’ represent a given variant for each SNP genotyped. Numbers in the columns marked “cases” and “controls” are the numbers of each class of genotype. Significant values (*p* < 0.05) are in bold.

# Continuity correction *P* value 0.001.

**Table 2:** Allele frequencies of DcR3 polymorphisms and their associations with breast cancer risk

SNPs of DcR3	Genotypes and Alleles	NO. (%)		OR (95% CI)	*P* value
		Cases (*n*= 531)	Controls (*n* = 592)		
Rs3208008 5′-near gene	A	724 (69.08%)	818 (70.03%)	Reference	
	C	324 (30.92%)	350 (29.97%)	1.046 (0.873–1.254)	0.627
Rs41309931 5′-near gene	G	785 (75.19%)	797 (68.94%)	Reference	
	T	259 (24.81%)	359 (31.06%)	***0.732 (0.607–0.884)***	0.001^*^
Rs2297441 5′-UTR	G	744 (71.54%)	800 (69.08%)	Reference	
	A	296 (28.46%)	358 (30.92%)	0.889 (0.740–1.068)	0.209
Rs1291207 Intron	A	729 (69.69%)	789 (67.78%)	Reference	
	G	317 (30.31%)	375 (32.22%)	0.915 (0.764–1.096)	0.334

* *P* < 0.01 (*P* = 0.0033) after correcting the *P* value for multiple testing by Haploview program using 10,000 permutations.

Rs3208008 cases *n* = 524, missing *n* = 7; controls *n* = 584, missing *n* = 8.

Rs41309931 cases *n* = 522, missing *n* = 9; controls *n* = 578, missing *n* = 14.

Rs2297441 cases *n* = 520, missing *n* = 11; controls *n* = 579, missing *n* = 13.

Rs1291207 cases *n* = 523, missing *n* = 8; controls *n* = 582, missing *n* = 10.

### Haplotype analysis

Haploview 4.1 analysis produced seven haplotypes with frequencies > 5% (Table 3). The most frequent haplotype that appeared in cases and controls was AGGA (rs3208008 A, rs41309931 G, rs2297441 G, rs1291207 A) (40.8%), but this was not significant. The frequency of haplotype ATGA (rs3208008 A, rs41309931 T, rs2297441 G, rs1291207 A) was significantly lower in cases compared with controls (*P* = 0.0053) and CGGA (rs3208008 C, rs41309931 C, rs2297441 G, rs1291207 A) had a higher frequency in cases (*P* = 0.0061). The other haplotypes were not significant (*P* > 0.05).

**Table 3:** Haplotypes of DcR3 gene (Frequency more than 5%)

DcR3 Haplotypes				Frequency	Cases (*n* = 531)	Controls (*n* = 592)	*P* value	OR (Odd Ratios)	Permutation *P* value^*^
S1	S2	S3	S4						
A	G	G	A	0.408	0.428	0.390	0.0693	1.211	0.1141
A	G	G	G	0.101	0.094	0.107	0.2951	0.863	0.9703
A	T	G	A	0.070	0.051	0.088	**0.0009**	**0.500**	**0.0053**
C	G	G	A	0.062	0.079	0.045	**0.0010**	**1.682**	**0.0061**
C	G	A	G	0.056	0.048	0.063	0.1398	0.756	0.7393
C	T	A	G	0.055	0.057	0.052	0.6271	1.131	1.0000
C	T	A	A	0.053	0.047	0.058	0.2626	0.789	0.9515

* After correcting the *P* value for multiple testing by Haploview program using 10,000 permutations.

S1 = rs3208008, S2 = rs41309931, S3 = rs2297441, S4 = rs1291207.

### Clinical features

We analyzed the relationships between DcR3 polymorphisms and a series of breast IDC clinicopathological features, including tumor size, lymph node metastasis and the statuses of ER, PR, C-erbB2 and P53. No DcR3 polymorphism was associated with tumor size. However, the frequencies of the AA genotype and A allele in rs2297441 were higher in lymph node metastasis positive cases (*P* = 0.030). The AG genotype in rs2297441 had a lower frequency in C-erbB2 positive cases (*P* = 0.033). In rs2297441, the AG and AA genotypes (*P* = 0.006 and 0.005, respectively) had lower frequencies in P53 positive cases and AA (*P* = 0.044) had a higher frequency in ER positive cases.

We found that the T allele in rs41309931 had a higher frequency in ER and PR positive cases (*P* = 0.042 and 0.045, respectively). The A allele in rs2297441 also had a higher frequency in lymph node metastasis, ER and PR positive cases (*P* = 0.035, 0.041 and 0.042), but had a lower frequency in P53 and C-erbB2 positive cases (*P* = 0.001 and 0.015). After correcting the *P-value* for multiple testing, we found that only the A allele in rs2297441 was significantly associated with P53 and C-erbB2 positivity (*P* = 0.0043 and 0.035, respectively).

We also analyzed associations between the haplotypes and clinical features. Haplotype AGGG (rs3208008 A, rs41309931 G, rs2297441 G, rs1291207 G) (7.7%) had a higher frequency in lymph node metastasis and P53 positive cases (*P* = 0.0309 and 0.0191). Haplotype AGGG (rs3208008 A, rs41309931 G, rs2297441 G, rs1291207 G) (7.7%) had a lower frequency in C-erbB2 and ER positive cases (*P* = 0.0088 and 0.0483). CTGA (rs3208008 C, rs41309931 T, rs2297441 G, rs1291207 A) had a lower frequency in PR positive cases. After correcting for multiple testing, only the AGGG haplotype in lymph node metastasis positive cases was significant (*P* = 0.0467), and no associations were observed between haplotypes and P53, C-erbB2, ER or PR status (*P* > 0.05).

## DISCUSSION

Breast IDC is the most common breast cancer type in women. Previous research concerning DcR3 polymorphisms is scarce [19, 20], especially with respect to cancer [21]. DcR3, as a member of the tumor necrosis factor superfamily of co-stimulatory molecules, promotes cellular invasion and migration in MCF7 breast cancer cells, and may be a negative regulator for aggressiveness during breast cancer development and progression [14]. We chose six SNPs with minor frequencies of more than 5% across the DcR3 gene region in the CHB population. Because the breast cancer DNA samples available for our research were not sufficient, only four of the DcR3 SNP loci could be analyzed statistically. In this study, rs41309931, located in the 5′-near gene region, may increase breast IDC susceptibility in Chinese Han females.

The 5′-near gene region is important for post-transcriptional modification and translation efficiency [22], and our work suggests that the rs41309931 DcR3 SNP in this region may function in breast cancer susceptibility. However, we found no association with susceptibility for rs3208008, which is also located in the 5′-near gene region. Additionally, introns perform vital roles in gene transcription and RNA stability. Mutations in introns can induce aberrant gene splicing due to disruption of splice site components, such as enhancers or silencers, or alteration of the mRNA secondary structure [23]. We found no associations between DcR3 gene intron polymorphisms and breast IDC. The promoter SNP locus rs2297441, in the Barbie Box and Dof2 binding site, could affect the binding of elements that regulate cell cycle-dependent DcR3 gene transcription. However, we again found no association between this genotype or allele distribution and breast IDC.

We also analyzed haplotypes of SNPs in the DcR3 gene using the Haploview program. The AGGA haplotype (rs3208008 A, rs41309931 G, rs2297441 G, rs1291207 A) exhibited the highest frequency of all haplotypes in the DcR3 gene, but was not significant. Haplotype ATGA (rs3208008 A, rs41309931 T, rs2297441 G, rs1291207 A) had a lower frequency in cases than controls and could thus be protective against breast IDC. Haplotype CGGA (rs3208008 C, rs41309931 G, rs2297441 G, rs1291207 A) had a higher frequency in cases than controls.

Steroid hormone receptors are valuable for predicting breast cancer prognosis and are regarded as predictive markers of endocrine therapy outcome [24]. Our results suggest that rs2297441 and rs41309931 may aid in breast IDC diagnosis and therapeutic decision-making. The oncogene, C-erbB2, and tumor suppressor, P53, play vital roles in breast cancer treatment and prognosis [25]; aberrant C-erbB2 expression and P53 mutation can lead to tumor metastasis, insensitivity to endocrine treatment and poor prognosis [26]. We found that the rs2297441 and rs41309931 polymorphisms were associated with P53 and C-erbB2 status. Thus, rs41309931 in the DcR3 5′- near gene region and rs2297441 in the promoter may be important in forecasting patient prognosis and tumor response to hormonal treatment. Lymph node metastasis can also serve as a prognostic indicator in breast cancer [27]. However, our study found that only the AA genotype and A allele in rs2297441 were notable with respect to lymph node metastasis. We also found that the AGGG (rs3208008 A, rs41309931 G rs2297441 G, rs1291207 G) haplotype was associated with lymph node metastasis and may be important for breast IDC proximal and distal metastasis.

In previous research, DcR3 was thought to be as a possible cancer biomarker [28], but few studies focused on the relationship between DcR3 gene polymorphisms and cancer risk. To our knowledge, this is the first report to associate DcR3 SNPs with breast cancer risk. Because polymorphisms often vary among ethnic groups, additional studies are needed to clarify the relationships of DcR3 polymorphisms with breast cancer in diverse ethnic populations.

## MATERIALS AND METHODS

### Immunohistochemistry and western blotting

We collected 55 carcinoma and pericarcinomatous tissue samples from female breast infiltrating ductal carcinoma (IDC) patients with clear pathologic diagnoses. Paraffin-embedded samples were stained with purified mouse anti-human DcR3 primary IgG antibody (BioLegend) followed by the ImmunoCruz mouse LSAB Staining System (sc-2050, SANTA CRUZ BIOTECHNOLOGY). Images were screened using a Nikon Eclipse 80i Microscope. For each specimen, immunohistochemical (IHC) staining proportion and intensity scores were determined [29]. Immunostaining intensity was scored based on visual assessment (0 = none; 1 = weak; 2 = intermediate, 3 = strong). The proportion score represented the percentage of positively stained cells by microscopic examination (0 = none; 1 = less than 5%; 2 = 5–25%; 3 = 26–50%; 4 = 51–75%; 5 = more than 75%). Overall DcR3 expression in each sample was expressed as a histoscore that was the sum of the proportion score (0–5) and the intensity score (0–3) for a range of 0–8, with a maximum possible score of 8. Values were reported as mean ± SE. The Mann-Whitney *U* test was used to analyze differences in DcR3 expression between tumor and pericarcinomatous samples.

Three breast cancer cell lines, including MD-MBA-231, SKBR3 and T47D, along with human mammary epithelial cells (HMEC), were sourced from the breast cancer tissue bank of the Third Affiliated Hospital of Harbin Medical University. All samples were resuspended in lysis buffer and 2 mM PMSF (Solarbio, China). DcR3 expression was detected by immunoblot using β-actin as a housekeeping protein. Primary antibodies were purified mouse anti-human DcR3 IgG (10 μg per 5 ml antibody dilution, BioLegend) and mouse anti-human β-actin monoclonal antibody (1:4000, Proteintech). The secondary antibody was peroxidase-conjugated Affinipure goat anti-mouse IgG (1:1000, Proteintech). The Luminata Forte Western HRP Substrate (Milliipore) was applied in exposure.

### Study subjects

This study included 531 female breast IDC cases (average age 49.5 ± 9.9 years) and 592 normal controls (average age 45.4 ± 9.9 years). All breast IDC cases, which were definitively diagnosed histopathologically in 2009, were recruited from the Breast Surgery DNA bank of the Third Affiliated Hospital of Harbin Medical University. Clinical feature information included tumor size, lymph node metastasis, human epidermal growth factor receptor 2 (C-erbB2), estrogen receptor (ER), progesterone receptor (PR) and tumor protein 53 (P53) statuses (Table 4). The normal female controls, who had no history of malignancy or autoimmune disorders, were random volunteers from the Third Affiliated Hospital of Harbin Medical University and were matched to cancer patients by age and same Chinese area. Approval from the Third Affiliated Hospital of Harbin Medical University ethical board was obtained before research began, and all volunteers gave written informed consent.

**Table 4:** Clinicopathologic information of breast IDC patients

Clinicopathologic information	Case No. (percentage)
**Tumor Size(cm)**	
Less than 2	186(0.3503)
2 to 5	246(0.4633)
More than 5	30(0.5650)
Unknown	69(0.1299)
**LN involvement**	
Positive	273(0.5141)
Negative	198(0.3729)
Unknown	60(0.1130)
**ER**	
Positive	277(0.5217)
Negative	189(0.3559)
Unknown	65(0.1224)
**PR**	
Positive	327(0.6158)
Negative	137(0.2580)
Unknown	67(0.1262)
**P53**	
Positive	143(0.2693)
Negative	314(0.5913)
Unknown	74(0.1394)
**CerbB-2**	
Positive	171(0.3220)
Negative	292(0.5499)
Unknown	68(0.1281)

IDC infiltrative ductal carcinoma, LN lymph node, TZ tumor size, ER estrogen receptor, PR progesterone receptor.

### SNP selection

DcR3 gene SNPs were selected using the Hapmap (hapmap.ncbi.nlm.nih.gov/) and dbSNP databases (www.ncbi.nlm.nih.gov/projects/SNP/). We chose six DcR3 SNPs, including rs3208008 and rs41309931 in the 5′-near gene region, rs2297441 in the 5′-UTR, rs2257440 and rs909341 in exons and rs1291207 in an intron. The minor genotype frequency of the six SNPs was more than 5% in the CHB population.

### DNA extraction and genotyping

Whole blood genomic DNA was extracted using the Universal Genomic DNA Extraction Kit Ver. 3.0 (TaKaRa, Japan). All six SNPs were genotyped via polymerase chain reaction restriction fragment length polymorphism (PCR-RFLP) assay. The polymorphic region was amplified by PCR using a T-Gradient Thermoblock PCR System (Biometra, Germany) in a 50 μl reaction solution containing 0.5 μg genomic DNA, 5 μl 10 × PCR buffer (Mg^2+^ plus), 4 μl dNTPs, 5 U TaqDNA polymerase (TaKaRa, Japan) and 0.5 μl of each primer (Invitrogen, China). Primers and restriction enzymes for PCR-RFLP genotyping are shown in Table 5. Annealing temperatures were rs3208008 (56°C), rs41309931 (62.8°C), rs2297441 (58.5°C), rs2257440 (56.6°C), rs909341 (58°C) and rs1291207 (57°C). Random samples of each SNP were directly sequenced to confirm the accuracy of genotyping results.

**Table 5:** Primers and PCR programs for DcR3 PCR-RFLP genotyping

SNP	primer	restriction enzyme	PCR products length
Rs3208008	F: 5′- GTAGCTGACTCCTGAACCG-3′ R: 5′- GGTCGTCGTCTTGCTTATAG-3′	CspCI	417 bp
Rs41309931	F: 5′-AGGGTTCAGCATGTTTGTG-3′ R: 5′-ATCTTGCTCTGGGTCTTCC-3′	BanI	307 bp
Rs2297441	F: 5′-ACCCACCCAACAGAATAGGC-3′ R: 5′-AAGTACAGTGTCACATGGCAG-3′	BbvI	410 bp
Rs2257440	F: 5′-AAAGGAGGTGGCATGTCG-3′ R: 5′-AGAGGACGTTGCAGTAGCG-3′	HinP1I	363 bp
Rs909341	F: 5′- CGCTGGTTTCTGCTTGGAG-3′ R: 5′-GATTGCTGCCCACTTTCC-3′	BpmI	640 bp
Rs1291207	F: 5′- GGCCTGATGGTAACTCTCC-3′ R: 5′- AGAGAGAAATCCCTGTGCAG-3′	BsrBI	351 bp

### Statistical analysis

Genotype frequencies of all SNPs were determined for Hardy-Weinberg equilibrium (HWE) assessment. We selected four SNPs for which enough data was available to perform statistical analyses. We used Haploview 4.1 software to tag all common haplotypes and their frequencies in both breast IDC cases and controls. To correct for multiple testing bias, we ran 10,000 permutations to determine *P* values. Relationships between SNPs and breast IDC risk were assessed by odds ratio (OR) and 95% confidence interval (CI). Disease characteristics were compared in all patients using the chi-square test. Genotype frequencies determined using different models of inheritance (additive, dominant, recessive and homozygote comparison) were analyzed using the chi-square test. Allele, genotype and haplotype frequency distributions were compared using the chi-square test, with *P* < 0.05 representing statistical significance. Statistical analyses were performed using SPSS 16.0 software.

## CONCLUSIONS

According to our limited case–control study results, we found that DcR3 SNPs differ between breast IDC cases and controls in a Chinese population. We also investigated the associations between DcR3 gene polymorphisms and breast IDC clinical features, such as lymph node metastasis and the statuses of ER, PR, CerbB-2 and P53. We can conclude that DcR3 SNPs may be associated with breast IDC risk in females of Northeast China.

## SUPPLEMENTARY MATERIALS FIGURES


