# Supplementary material for: DcR3 gene polymorphisms are associated with sporadic breast infiltrating ductal carcinoma in Northeast Chinese women
Source: Oncotarget. 2016 Aug 9;7(36):57970–7. doi: 10.18632/oncotarget.11153 (PMC5295404; doi:10.18632/oncotarget.11153)
Supplement: Supplementary file 1 [file oncotarget-07-57970-s001.pdf]

DcR3 gene polymorphisms are associated with sporadic breast infiltrating ductal carcinoma in Northeast Chinese women

Supplementary Materials

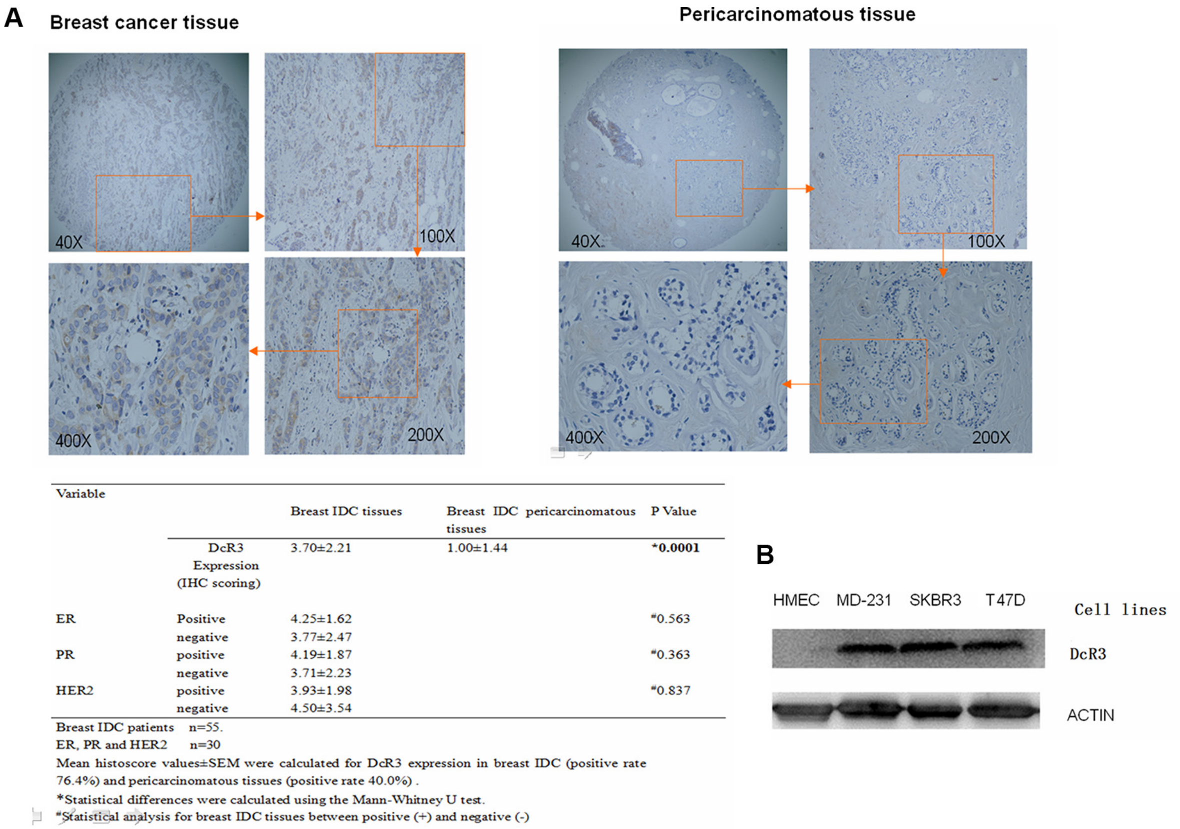

Variable		Breast IDC tissues	Breast IDC pericarcinomatous tissues	P Value
	DcR3 Expression (IHC scoring)	3.70±2.21	1.00±1.44	***0.0001**
ER	Positive	4.25±1.62		#0.563
	negative	3.77±2.47		
PR	positive	4.19±1.87		#0.363
	negative	3.71±2.23		
HER2	positive	3.93±1.98		#0.837
	negative	4.50±3.54		

Breast IDC patients n=55.
ER, PR and HER2 n=30
Mean histoscore values±SEM were calculated for DcR3 expression in breast IDC (positive rate 76.4%) and pericarcinomatous tissues (positive rate 40.0%) .
*Statistical differences were calculated using the Mann-Whitney U test.
#Statistical analysis for breast IDC tissues between positive (+) and negative (-)

Supplemental Figure S1: Evaluation of DcR3 expression in breast cancer. DcR3 was detected in paraffin-embedded tissue sections using IHC analysis (**A**). IHC staining scores are reported as means ± SE. The Mann-Whitney *U* test was used to analyze *DcR3* expression differences between IDC and pericarcinomatous (PC) tissues (3.70 ± 2.21 to 1.00 ± 1.44). $P = 0.0001$. DcR3 was detected in HMEC and breast cancer cell lines via western blotting (**B**). DcR3 is overexpressed in breast cancer cell lines as compared to HMECs. Experiments were repeated 4 times.
